# Quality assessment of an Emergency Department

**DOI:** 10.1186/1757-7241-20-S2-P42

**Published:** 2012-04-16

**Authors:** David Levarett Buck, Thomas Osterland, Thomas Andersen Schmidt, Søren Wistisen Rasmussen

**Affiliations:** 1 The Emergency Department, Holbæk Hospital, Holbæk, Denmark; 2 Quality and Development, Region Sjælland, Sorø, Denmark

## Background

Because many emergency departments (ED) have become independent wards, great focus has been put on efficiency.

The ED at Holbaek Hospital was established in 2009. The objective of this study was to evaluate the efficiency of the ED.

## Methods

We evaluated the following:

The percentage of mis-referrals to stationary wards, i.e. transferals to another specialty than the one chosen by the emergency department within 24 hours.

The percentage of re-admitted patients within 30 days after discharge.

For assessment we searched the National Patient Registry for all patients admitted to the ED during 2010 compared to 2008, i.e. the year before the ED was established.

## Results

Acute patients have increased with 19% from 2008 to 2010. Despite this, approximately 800 fewer patients (9%) were admitted to the medical wards and 300 (17%) fewer to the orthopedic wards in 2010.

Of the 33,127 patient contacts in our ED during 2010, only 12,710 (38.4%) were admitted to a stationary ward for further diagnostics and treatment, because definitive treatment was initiated in the ED. The majority of these (95%) were not transferred to another specialty within 24 hours.

There has been an increase in patients discharged after 1-2 days of 6.7%, and a decrease in the length of stay at stationary wards by 12.7% to 3.45 days from 2008 to 2010.

Nevertheless, the frequency of re-admitted patients within 30 days was diminished by 20.3%, amounting only 8.8% in 2010 (the national average being 9.3%).

## Conclusion

Since the opening of our ED, there has been a general increase in acute patients of 19%. Despite that, there has been a noticeable reduction in patients admitted to the medical and orthopaedic wards. The vast majority of patients are admitted to the correct ward by the emergency physicians, indicating very few mis-referrals. The ED appears to initiate proper diagnostics and treatment, hence leading to a shorter length of stay at stationary wards. Despite a decrease in the length of stay by 12.7%, the frequency of re-admitted patients has been reduced with 20.3% after the opening of the ED, and is 0.5 percentage points lower than the national average.

